# User Evaluation of a Shared Robot Control System Combining BCI and Eye Tracking in a Portable Augmented Reality User Interface

**DOI:** 10.3390/s24165253

**Published:** 2024-08-14

**Authors:** Arnau Dillen, Mohsen Omidi, Fakhreddine Ghaffari, Olivier Romain, Bram Vanderborght, Bart Roelands, Ann Nowé, Kevin De Pauw

**Affiliations:** 1 Human Physiology and Sports Physiotherapy Research Group, Vrije Universiteit Brussel, 1050 Brussels, Belgium; 2 Equipes Traitement de l’Information et Systèmes, UMR 8051, CY Cergy Paris Université, École Nationale Supérieure de l’Electronique et de ses Applications (ENSEA), Centre National de la Recherche Scientifique (CNRS), 95000 Cergy, France; fakhreddine.ghaffari@cyu.fr (F.G.); olivier.romain@cyu.fr (O.R.); 3 Brussels Human Robotics Research Center (BruBotics), Vrije Universiteit Brussel, 1050 Brussels, Belgium; mohsen.omidi@vub.be (M.O.); bram.vanderborght@vub.be (B.V.); 4 IMEC, 1050 Brussels, Belgium; 5 Artificial Intelligence Lab, Vrije Universiteit Brussel, 1050 Brussels, Belgium

**Keywords:** brain-computer interface, human-robot interaction, user evaluation, usability, assistive robotics, augmented reality, shared control, user experience

## Abstract

This study evaluates an innovative control approach to assistive robotics by integrating brain–computer interface (BCI) technology and eye tracking into a shared control system for a mobile augmented reality user interface. Aimed at enhancing the autonomy of individuals with physical disabilities, particularly those with impaired motor function due to conditions such as stroke, the system utilizes BCI to interpret user intentions from electroencephalography signals and eye tracking to identify the object of focus, thus refining control commands. This integration seeks to create a more intuitive and responsive assistive robot control strategy. The real-world usability was evaluated, demonstrating significant potential to improve autonomy for individuals with severe motor impairments. The control system was compared with an eye-tracking-based alternative to identify areas needing improvement. Although BCI achieved an acceptable success rate of 0.83 in the final phase, eye tracking was more effective with a perfect success rate and consistently lower completion times (p<0.001). The user experience responses favored eye tracking in 11 out of 26 questions, with no significant differences in the remaining questions, and subjective fatigue was higher with BCI use (p=0.04). While BCI performance lagged behind eye tracking, the user evaluation supports the validity of our control strategy, showing that it could be deployed in real-world conditions and suggesting a pathway for further advancements.

## 1. Introduction

Physically assistive robots aim to enhance the autonomy of individuals with physical disabilities by enabling interaction with their environment through the robot [1]. In the European Union, there are 9.53 million stroke survivors, often resulting in impaired motor function, and an additional 2.58 million stroke cases are predicted by 2047 [2]. Thus, there is an urgent need for assistive technologies that enable motor-impaired individuals to interact with their surroundings. An ideal assistive robot should anticipate the user’s intentions while avoiding unintended actions. This requires an interaction modality that monitors the user’s attention and infers their intentions from physiological behavior.

One promising interaction modality for addressing motor function impairments is the brain–computer interface (BCI). Formally defined by [3], “A BCI is a system that measures central nervous system (CNS) activity and converts it into artificial output that replaces restores, enhances, supplements, or improves natural CNS output and thereby changes the ongoing interactions between the CNS and its external or internal environment”. In the context of human–computer interaction, a BCI is defined as a system that allows users to operate devices with their thoughts by measuring and decoding neural activity.

BCI control systems interpret neural activity from brain-related physiological signals, such as the electroencephalogram (EEG), by applying machine learning (ML) techniques trained on labeled data from individuals engaged in mental tasks [4]. In BCI control systems, classification is used alongside high-level control software to convert decoded mental tasks into device actions. Various modalities of the EEG signal are utilized for BCI control [5], with one commonly used paradigm being motor imagery (MI). MI refers to the mental process of imagining a movement without physically executing it [6], defined as “the mental simulation of an action without the corresponding motor output” [7]. A BCI system employing MI decodes the imagined movement from the EEG signal and executes a command corresponding to the decoded class.

Currently, BCI technologies are primarily confined to lab settings where environmental conditions are controlled, and distractions are minimized. Before BCI can be used in real-world applications, several key issues need resolving [8]. Developing a BCI control system with a good user experience requires a user-centered design approach for the control strategy and user interface (UI) [9]. This approach has gained traction within the BCI community [10,11,12], although most BCI research focuses only on technical validation [13].

A key component of a user-centered approach is evaluating the control system with users in realistic scenarios to ensure its performance under real-world conditions. Assessing the real-world readiness of a BCI control system requires appropriate performance measures to quantify its usability [14]. A comprehensive usability assessment should encompass effectiveness, efficiency, and user experience through both objective and subjective measures [15].

Objective measures should include decoding-related metrics such as accuracy, as well as performance metrics for task completions, including completion time and success rate [10]. Subjective measures can be collected through qualitative interviews and quantitative metrics that facilitate comparisons between different control systems or versions of the same system. Tools like visual analog scales and questionnaires can provide quantitative user experience metrics [16]. To ensure thorough user studies, it is crucial to establish standard guidelines and an evaluation framework that supports the user assessment of novel BCI control systems. The recent efforts to standardize BCI usability research are ongoing [17,18], but more work is still required to facilitate the usability evaluation of BCI prototypes.

The user study described in this article aims to evaluate the real-world usability of a BCI control system developed in the previous research. The central research question is to evaluate whether our MI-based BCI control system prototype could be deployed in real-world applications. The study involved able-bodied participants using the BCI control system to operate a robotic arm in tasks representative of daily activities. Additionally, the BCI control system was compared with an alternative eye-tracking-based control system to assess the added value of BCI in everyday scenarios for a physically assistive robot. The results are intended to provide insights into areas needing improvement and quantify the usability of the BCI control system, also providing a framework for other BCI researchers to evaluate their control system prototypes.

## 2. Related Work

Most BCI user studies primarily address the effectiveness and efficiency aspects of usability [18,19,20]. An exemplary study that thoroughly evaluates all aspects of usability was conducted by Kuhner et al. [21], who present an extensive experiment with their prototype. Usability studies can vary in focus depending on the application and method being investigated.

Some studies explicitly compare different control paradigms [22,23], while others aim to validate the technical feasibility of a new prototype before proceeding to more comprehensive user studies or clinical trials [24,25,26]. User studies may involve clinical trials with specific patient populations [27,28,29,30] or studies with able-bodied individuals that focus on the user experience of a new approach [31,32]. Usability is also crucial when developing new hardware [25]. The review by Ortega and Mezura-Godoy [33] offers a recent systematic overview of BCI user evaluation literature.

## 3. Materials and Methods

### 3.1. The BCI Control System

#### 3.1.1. Control Strategy

**Figure 1:**
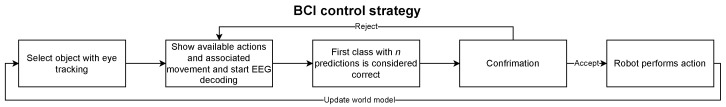
The control strategy of the MI BCI control system. The user selects an object with their gaze and uses MI to select one of the possible actions. After accepting or rejecting the decoded MI class, the robot executes the associated action or returns to the action selection stage.

The BCI control system that is evaluated in this user study uses a shared control approach, which is depicted in Figure 1.

The control strategy involves several stages, beginning with object selection. To interact with an object in the environment, the user gazes at the chosen object for 2 s, prompting the system to advance to the action selection stage. Here, possible actions appear as a menu above the object, indicating the specific movement the user should imagine in order to select an action. The shared control system utilizes an internal world model, updated by sensors such as cameras, to identify the object’s type, location, and state. This allows the system to determine which actions are available to the user.

Next, real-time EEG decoding initiates, putting the system in the decoding stage. In this stage, the user imagines one of the movements associated with a robot action, continuing to do so until the EEG decoding software makes *n* predictions for the same MI class. The prediction threshold was set to 5 for this study. Once the decoding threshold is met, the system transitions to the confirmation stage, where the user is presented with a holographic modal window showing the action that was determined and asked to accept or reject it.

If the user accepts the decoding result, the robot executes the corresponding action. If the user rejects the result, decoding resumes, and the user imagines the intended movement again. This method prevents the robot from performing unintended actions, avoiding the need for the user to cancel an action during its execution. After the robot completes the selected action, the system returns to object selection mode. The movement inferred by the control system is referred to as a decision, as opposed to a prediction from the decoding pipeline for a single window of EEG. We use the term decision hereafter to refer to the expected action resulting from several MI predictions.

An eye-tracking-based variant was implemented to compare the usability of the BCI control system with an alternative control modality. The object selection strategy mirrors that of the BCI control system. However, in the eye-tracking variant, the menu displaying possible actions consists of buttons that are selectable via eye tracking. The associated robot action is executed when the user gazes at an action button for 2 s. Thus, the decoding and confirmation stages are eliminated from the control strategy, made possible by the near-perfect accuracy of eye tracking.

The UI software that handles the logic of the decoding strategy and the contextual display of graphical components, such as menus and virtual evaluation environments, was implemented using the Unity 3D [34] game engine and the mixed reality toolkit version 2.8 [35]. Communication with the real-time EEG decoding process, which is detailed in Section 3.1.3, is achieved with a publish/subscribe strategy. The UI process sends messages to start and stop decoding to the EEG decoding process and the EEG decoding process sends decoding results back to the UI.

#### 3.1.2. Hardware

Two EEG devices were used in this user study. Initially, the OpenBCI EEG device with a 16-electrode cap was employed for the first three participants in Phase 1. This device uses passive gel-based Ag/AgCl electrodes, samples at 125 Hz, and transmits data wirelessly via Bluetooth. Subsequently, the Smarting ProX (mBrainTrain, Belgrade, Serbia) was used, featuring a 64-electrode cap with active gel-based electrodes. The sampling rate was set to 250 Hz in this study to reduce computational costs for real-time processing. The Smarting ProX was used from Phase 2 onward to allow for more detailed analysis of the EEG data in future research, such as investigating cognitive processes related to MI. The OpenBCI device was initially used to demonstrate the feasibility of using consumer-grade EEG with our control system. Both electrode layouts follow the international 10–20 system [36].

Microsoft^™^ HoloLens 2.0 was chosen for the head-mounted AR display. It uses waveguide-based AR and provides off-the-shelf eye-tracking and spatial awareness functionality through its built-in sensors. This device enabled the AR display of the UI overlaid on the real world while also providing the necessary sensor information for the shared control strategy. The Franka Research robot (Franka Robotics GmbH, Munich, Germany) was employed as the robot arm in this experiment. This robotic arm offers 7 degrees of freedom and is equipped with a gripper to pick up objects [37].

Real-time EEG decoding was performed by a laptop equipped with a 6-core Intel(R) Core(TM) i7-10850H CPU running at 2.70 GHz. The laptop has 16 GB of RAM and an NVIDIA GeForce RTX 2070 Max-Q GPU. It connects to the robot via Ethernet and communicates with the HoloLens through a USB-C cable.

#### 3.1.3. Real-Time EEG Decoding

The real-time EEG decoding component of the control system was implemented with the Python 3.10 programming language. The MNE library [38] was used for operations relating to EEG data processing such as reading data files, filtering, and epoching, among others. MNE was also used to implement the feature extraction step of our decoding pipeline. The lab streaming layer [39] software was used with its Python client PyLSL to integrate the real-time EEG data stream with our software. The Scikit Learn (SKLearn) library [40] was used for the implementation of ML models and decoding pipeline management.

When the decoding stage begins, the process starts by sampling chunks of EEG data from the EEG data stream. For this study, each chunk is 4 s long, meaning that the most recent 4 s of EEG data are sampled. Multiple windows of EEG data are then extracted from each chunk using a sliding window approach with a stride of 0.25 s. Each input window is 2 s long, resulting in 9 overlapping windows per chunk. These windows are subsequently sent to the decoding pipeline for classification.

The first step in the decoding pipeline involves filtering the EEG data using a finite impulse response band-pass filter. The filter design is a windowed time-domain (firwin) design, as recommended by Widmann et al. [41]. This filter is a one-pass, zero-phase, non-causal band-pass filter using a Hamming window with 0.0194 passband ripple and 53 dB stopband attenuation. The lower passband edge is set at 8 Hz with a 2 Hz transition bandwidth for a −6 dB cutoff frequency at 7 Hz. The upper passband edge is at 30 Hz with a 7.5 Hz transition bandwidth for a −6 dB cutoff frequency at 33.75 Hz. The filter length is automatically set to 413 samples (1.652 seconds) based on the default settings of the MNE filter functions.

After filtering, features are extracted from the input windows using common spatial patterns [42], with 6 components utilized in this step. These features are then used to predict the MI class using a linear discriminant analysis model [43]. The decoding pipeline was trained on calibration data that were previously acquired according to the procedure detailed in Section 3.2.3. The decoding pipeline was always trained with all the calibration data that were acquired during the current session and never used data from other sessions.

### 3.2. User Study

The real-world usability of the prototype control system was assessed through a user study where participants operated a virtual robotic arm using our control system. The study was divided into three distinct phases to allow iterative improvements of the software, ensuring a stable experience for the final phase. Participants completed various scenarios representing everyday chores in a virtual AR environment. The evaluation tasks utilize holograms to simulate the robot arm and objects within a real-world environment. In the final session of Phase 3, a real robot is incorporated to replicate the actions performed by the virtual robot. The procedure was consistent across phases, differing only in the number of sessions and the tasks to be completed.

New participants were recruited for each phase to assess the experience of users unfamiliar with BCI and to improve the control system between phases. The number of participants was increased with each phase to ensure rapid iteration while maintaining the validity of the results. The sample sizes were determined based on best practices in the literature [10,13,44].

In Phase 1, a validation study was conducted using the OpenBCI EEG headset, involving 3 participants (all male, aged 23–24), each attending 2 sessions. Phase 2 involved 5 participants (1 female, 4 male, aged 23–29), each attending 3 sessions. This phase allowed for validation of the procedure and further technical validation of the system. Since the statistical comparison of outcomes was not required at this stage, 5 participants provided a good balance between thorough assessment and time efficiency. The performance assessment used the same sorting task as in Phase 1, with the main difference being that participants had to complete 10 repetitions of the sorting task to consider a run successful.

In the final phase, the procedure was expanded by introducing a pick-and-place task and using a real robot in each participant’s final session. This phase involved 12 participants (3 female, 9 male, aged 22–30), each attending 3 sessions. The sample size was determined using established usability engineering guidelines [14,15]. The procedure for each session is detailed in Section 3.2.3.

The participants received an explanation of the procedure upon registration and provided written informed consent at the start of the first session. The instructions were orally given before each run of a usage scenario. This study was approved by the Medical Ethics Committee of UZ Brussel and VUB (BUN1432023000232) and adhered to the principles of the Declaration of Helsinki for medical research involving human participants [45].

The questionnaire results were collected using the Redcap software version 14.1.0 [46]. The result data were processed with the Python programming language using the Pandas library for organizing the data [47] and the Seaborn library for generating figures [48]. The statistical tests presented in this study were performed using the stats package of the SciPy library [49].

#### 3.2.1. Evaluation Scenarios

The first evaluation scenario involves sorting a cube using the robotic arm, simulating everyday tasks such as sorting garbage or laundry. In this scenario, the participant is seated and equipped with EEG and AR devices. A virtual robot arm is placed on a virtual table in front of them, with two differently colored baskets on either side. A colored cube appears before the participant, indicating which basket to select based on the cube’s color. The user must choose the correct side by following instructions from the action menu that appears after looking at the cube for 2 s. Each run includes multiple repetitions of the task until the specified end conditions, detailed in Section 3.2.3, are met.

The second task is a pick-and-place scenario where the participant, still seated and equipped with EEG and AR devices, interacts with the virtual robot arm and four virtual objects on the table: an orange, a Rubik’s cube, a TV remote, and a smartphone. The objects are placed at fixed positions and the possible actions always consist of instructing the robot to pick up the object and either give it to the user (simulated by placing the object at a fixed position in front of the user) or put it away at an object-dependent fixed position. When the real robot is connected, the virtual objects are represented by cubes placed in a fixed location on the table the robot is placed on. The participant completes a fixed sequence of actions that was determined beforehand to complete the run. The order of objects and actions is randomized for each run.

#### 3.2.2. Usability Measures

To assess the control system’s objective performance, several metrics were used. The main performance metric of this study is the success rate for task completion, calculated as

(1) $$success-rate=\frac{n_{c}}{n_{r}},$$

where $n_{c}$ is the number of completed runs and $n_{r}$ is the total number of runs performed. This metric represents the system’s effectiveness in enabling users to complete their assigned tasks using the control system.

The efficiency of the control system is measured by the task completion time. The completion time is calculated as the time between the start signal and placing the final object in its intended location.

To assess the online decoding performance of our decoding pipeline, we compute the decision accuracy based on rejected and accepted actions during the final evaluation runs of participants. We chose balanced accuracy as our prediction performance measure, which is computed as

(2) $$balanced-accuracy=\frac{1}{2}\left(\frac{TP}{TP+FN}+\frac{TN}{TN+FP}\right)$$

where TP is the number of true positives, FN is the number of false negatives, TN is the number of true negatives, and FP is the number of false positives. When referring to accuracy in the remainder of this manuscript, balanced accuracy is implied.

The often-used user experience questionnaire (UEQ; [50]) and a semi-structured interview were used to assess the user experience of the system quantitatively and qualitatively, respectively. The UEQ questionnaire asks participants to give their opinion regarding several concepts on a scale from 1 to 7. The questions for the user interview can be found in Appendix A.

To assess the amount of subjective fatigue that is induced by the control system, a visual analog scale was used where users had to indicate their perceived mental (mVAS) and physical (pVAS) fatigue levels [51]. The indicated VAS levels were subsequently discretized to scores ranging from 0 to 100. The VAS approach was chosen for its short application time and easy explainability to participants. Subjective fatigue was chosen as opposed to more objective measures, such as features from the EEG signal, as we are mostly interested in user perception. The participant’s mood state was also assessed using the profile of mood states (POMS) questionnaire [52]. The participant’s aptitude for MI is evaluated with the motor imagery questionnaire 3 (MIQ3; [53]).

We compared the performance outcomes of the control system variants using statistical testing. The significance level was set to 0.05 for all tests. The data were first checked for normality using D’Agostino and Pearson’s test [54]. For paired samples, such as UEQ responses between BCI and eye tracking and fatigue levels before or after using the control system, paired tests were used. If normality was ensured, a paired t-test [55] was used to investigate if there was a statistical difference between the mean scores for BCI and eye tracking. The Wilcoxon signed-rank test [56] was used if the normality of the data was rejected.

When the data were independent, such as when comparing eye tracking and BCI mVAS outcomes from the final evaluation session (since the order of the control system variant was alternated for participants), independent sample tests were used. When the data were normally distributed, an independent samples t-test was used [55] and the Mann–Whitney U test [57] was chosen if this was not the case.

#### 3.2.3. Procedure

Each phase consisted of multiple sessions where the first sessions were intended to support participant’s familiarization with the concept of MI BCI and the interaction design of the control system. During these sessions, participants were encouraged to ask questions if something was unclear while completing a task and were frequently questioned about their experience. The first session always started with signing the informed consent after a briefing and giving the participant time to read the document in detail. This was then followed by the MIQ3 questionnaire, after which participants were requested to complete the POMS, mVAS, and pVAS questionnaires. Participants were instructed to complete the latter three questionnaires at the start of each subsequent session and to fill out the pVAS and mVAS again at the end of every session.

**Figure 2:**
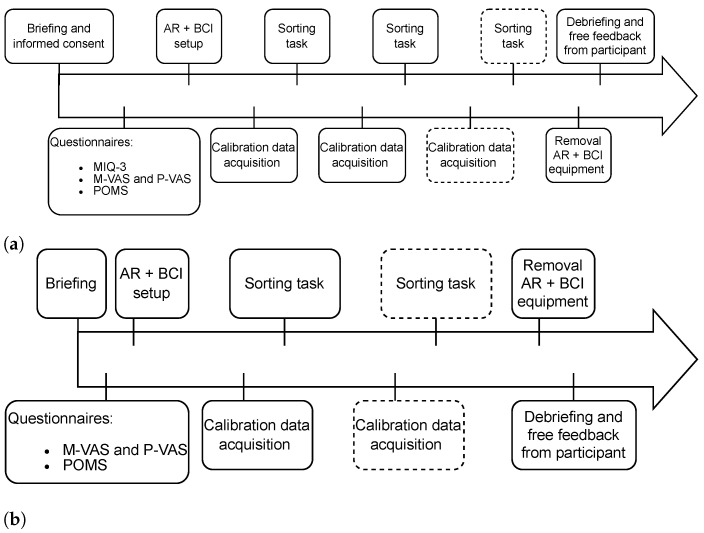
The experimental procedure that was followed for Phase1 in (**a**) session 1 and (**b**) session 2.

Ensuing sessions focused on training the participant in using the control system with the last session culminating in a performance assessment employing one of the evaluation tasks. After completing the questionnaires, the acquisition of calibration data that were used to train an MI decoding pipeline ensued. The subsequent tasks were dependent on the study phase and session number. Figure 2 shows an overview of the procedure for Phase 1, highlighting the differences between the sessions.

In Phase 1, participants performed the sorting task with the robot. After filling in the questionnaire and setting up the EEG and AR devices, calibration and sorting runs were alternated. There was no fixed amount of trials or runs as this phase was merely intended to validate the technical feasibility of using our control system with OpenBCI. The session ended when the planned 3-h time limit was reached or the participant decided that they were satisfied with their performance, having attempted at least two sorting runs. The second session followed the same procedure as the first with the difference that informed consent and MIQ3 are omitted and only 1 run is required to complete the session.

**Figure 3:**
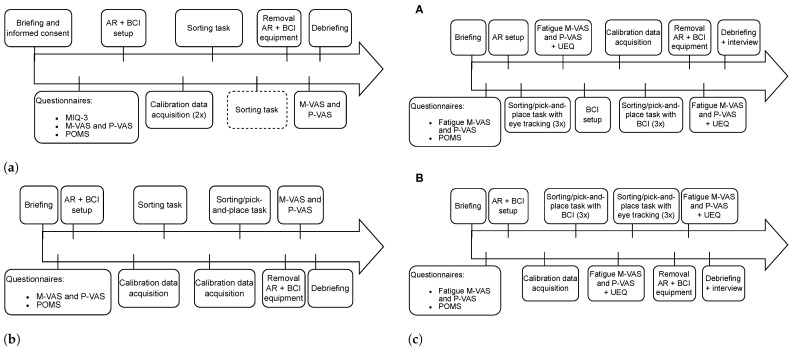
The experimental procedure that was followed for Phase 3 in (**a**) session 1, (**b**) session 2, and (**c**) session 3 with options **A** and **B**.

Phases 2 and 3 use the same procedure with some key differences in the evaluation tasks that are used. The order of session events is exactly the same. Figure 3 shows the procedure for each session with Figure 3c showing variants of session 3, labeled as A (eye tracking first) and B (BCI first).

For the first session of Phase 2, shown in Figure 3a, participants completed two consecutive calibration runs with a 5-min break in between. Subsequently, they were requested to perform one sorting run with the possibility of an optional second run if there was time and they were willing. The second session, depicted in Figure 3b, consisted of an alternation of calibration and sorting runs with a maximum of 2 calibration runs. Participants were encouraged to perform a third sorting run if possible. There was no time limit for the first two sessions.

In session 3, illustrated in Figure 3c, the participant had to perform three consecutive sorting runs that were timed using either the BCI or eye tracking control system variant. A calibration run was performed beforehand if the BCI variant was used first (B). If participants used eye tracking first (A), we performed the EEG setup and calibration run after they completed the evaluation runs with eye tracking. The time limit was set to 15 min and a run was considered failed if the participant was unable to complete the tasks within this limit. After completing the required runs, the participant was requested to complete the mVAS and pVAS to assess their intermediate fatigue levels.

This was followed by the UEQ questionnaire in relation to their experience with the used control system. Afterward, following the necessary setup and eventual BCI calibration, the participant performed 3 runs with the other control system variant. This was again followed by the mVAS, pVAS, and UEQ questionnaires in relation to the current variant. The order of variants used was alternated between participants to avoid possible bias resulting from the participant’s experience with the other variant and to investigate the change in fatigue levels compared to the baseline at the beginning of the session. Finally, the interview was performed as a debriefing to assess if participants had suggestions.

The first session of Phase 3 was identical to Phase 2, and the second session also started with a calibration run followed by a sorting run. After the second calibration run, the pick-and-place task was introduced. Participants were asked to choose the order of objects and the actions themselves. They had to announce their choice before initiating the object selection stage of the control system. Afterward, they were encouraged to perform an optional run where the experimenter provided the sequence of object–action pairs to prepare them for the final session.

Finally, the last session introduced the real robot and took place at the AI Experience Center at VUB, located in Brussels, Belgium, where the robot arm is located. This resulted in more noisy, although more realistic, conditions. The pick-and-place task was used for all evaluation runs of this session.

## 4. Results

**Figure 4:**
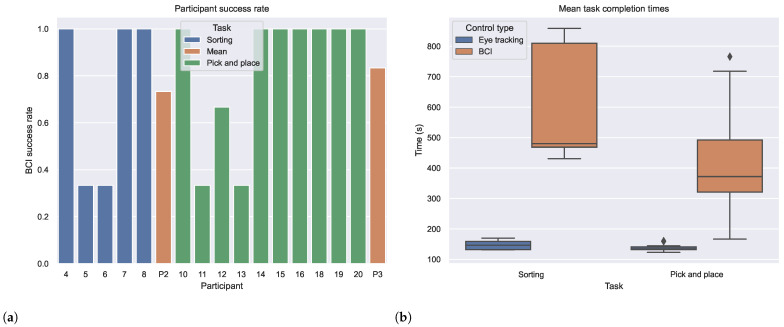
(**a**) Success rate for individual participants together with the mean for each phase and (**b**) boxplots comparing the mean completion times between the eye tracking and BCI control system variants for each task.

The success rate in completing the evaluation tasks with BCI in the final session for each participant is shown in Figure 4a, together with the mean value over each phase. The performance metrics were only recorded in Phases 2 and 3 and data are missing for participants 9 and 17 due to a technical issue resulting in the loss of timings for one or more runs of the last session. Figure 4b displays boxplots comparing the eye tracking and BCI control system variants for both tasks.

We can observe that during both phases, most users achieved a perfect success rate. However, participants who did not reach a perfect success rate experienced runs where the system repeatedly made incorrect predictions, which significantly impacted their motivation and caused frustration. Notably, participants 11 and 13 exhibited high baseline fatigue levels at the start of the session, with mVAS scores of 72 and 55, respectively. Additionally, participant 12’s final session was disrupted by a fire drill, likely affecting their ability to regain focus and maintain an appropriate mental state. The mean success rates for phases 2 and 3 were 0.73 and 0.83, respectively. In contrast, the success rate for eye tracking was consistently 1 for all participants.

Figure 4b illustrates that completion time is longer when using the BCI control system compared to the eye tracking variant. The variance is also significantly higher for BCI, indicating less consistent efficiency. Despite the mean completion time for BCI being significantly higher (p<0.001), some participants were able to nearly match the eye tracking performance in the pick-and-place task during Phase 3. The lower efficiency of BCI can be attributed to the added confirmation step and its lower accuracy. While eye tracking achieved a perfect recognition rate, BCI’s online decoding accuracy was only 0.52. However, this was offset by our decision strategy, resulting in the observed success rates.

The mean results for each question in the UEQ questionnaire are presented in Table 1. The left term in the Question column represents the left side of the scale and corresponds to a value of 1 while the right term corresponds with a value of 7.

**Figure 5:**
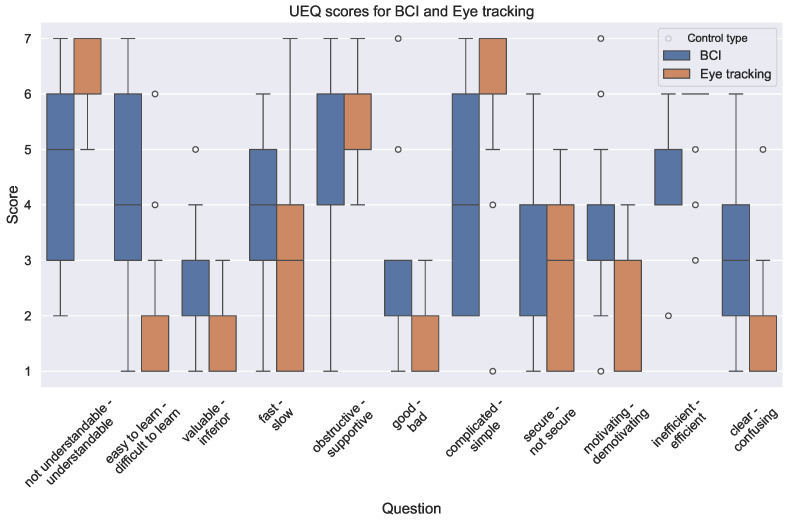
UEQ questions where a significant difference was found between the participant’s answers for the eye tracking and BCI variants. A score of 1 indicates that users felt more that the top term of the label was applicable, while 7 means that the bottom term was more applicable.

For most questions in the UEQ, there is no statistically significant difference in the user’s experience. This indicates that the user experience was mostly similar regarding these aspects of the control system. The aspects where significant differences were found are shown in Figure 5 for a closer investigation.

We observe that all differences favor the eye tracking variant and that the questions pertain to efficiency and perceived complexity. In most cases, except *fast—slow*, *secure—not secure*, and *motivating—demotivating*, the variance is larger for BCI than for eye tracking. This indicates that the experience of participants was more variable when using BCI, which can be related to the variance in success rate and completion time that was observed in the objective performance metrics. We also note that there was a large agreement that eye tracking was efficient, as the majority of participants scored 6, with only 4 participants giving another score.

**Figure 6:**
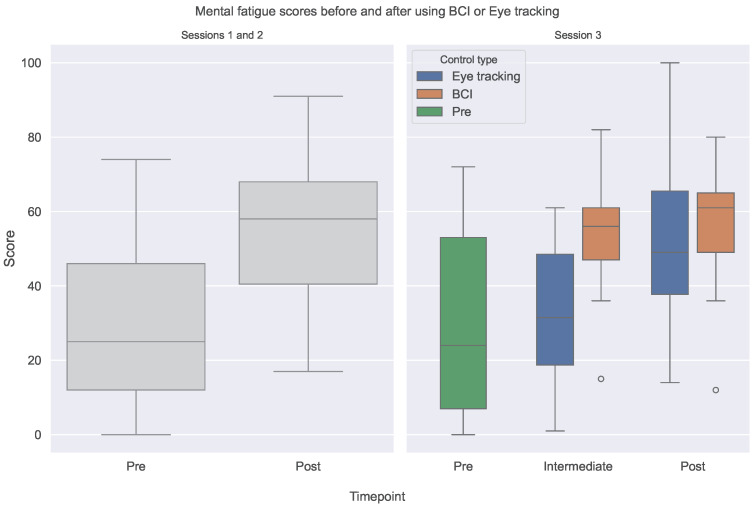
mVAS scores at the beginning of each session and the end for the first two sessions and for sessions 3 before, between the first and second evaluation rounds, and at the end of the session, split by the control system that was used.

Finally, we investigate the changes in mental fatigue entrained by the control systems. Figure 6 shows the difference in mVAS scores between the start of the session and the end for the first two sessions and between the beginning, the intermediate, and final fatigue assessments, split by control system type, used for session 3.

We can observe a significant increase in fatigue between the beginning and the end of a session (p<0.001) when analyzing the session 1 and 2 plots. This shows that the calibration procedure followed by using the BCI control system induces significant mental fatigue. From the session 3 results, we notice that the intermediate result for BCI is significantly higher (p=0.04), while the difference is lower, and not significant anymore (p=0.78) at the end of the session.

## 5. Discussion

This study aimed to evaluate the real-world usability of a BCI control system designed to operate an assistive robotic arm for physically incapacitated users. The system employs a shared control approach, combining BCI with eye tracking in a mobile AR UI. To balance the need for rapid software development [58] with system stability during user studies [59], the evaluation was divided into three phases. To assess the additional value provided by BCI and establish a performance benchmark, the study compared the BCI control system with a version using only eye tracking to navigate the system UI.

The first phase of the user study demonstrated the feasibility of using consumer-grade EEG devices for BCI control with our control strategy. This finding, along with the previous research [60], suggests the potential for using low-cost EEG devices in real-world BCI applications. Such advancements could make BCI technology more accessible to consumers, paving the way for commercial applications.

In Phases 2 and 3, we found that eye tracking outperformed BCI in all aspects of usability. All participants successfully completed the evaluation tasks in the final session using eye tracking, whereas the success rates were only 73% and 83% for Phases 2 and 3, respectively, when using BCI. The performance gap between the two methods was even more evident when comparing task completion times. Although these issues were previously recognized [6], our study quantifies these discrepancies and sets a performance benchmark for BCI to be considered a viable alternative to eye tracking for navigating an AR UI.

While most participants successfully completed all evaluation runs, some exhibited poorer task completion performance. This underperformance is likely due to participants arriving fatigued for the experiment, as fatigue is known to diminish the reliability of MI performance [61]. Additionally, effective MI requires sustained focus, which can be compromised by frustration from repeated failures or external distractions [62]. The poor performers all exhibited high baseline fatigue levels or were distracted by external events, such as one participant who experienced a fire drill during the experiment.

In terms of user experience, the differences were fewer and smaller, but participants showed a significant preference for eye tracking for some aspects. Despite this, several participants expressed during interviews at the end of the final session that they would prefer BCI if it could be made more reliable. The aspects where eye tracking performed significantly better were related to complexity and efficiency, which are primarily engineering issues that can be addressed with existing technologies [63,64] and a user-centered UI design [12].

Despite the challenge of comparing our usability outcomes with the existing literature due to the diversity of evaluation methods and control strategies, we can make high-level comparisons with similar studies. Our decision accuracy aligns with the expected decoding accuracy for the models used [65,66]. Additionally, our task completion performance is consistent with previous studies employing a shared control strategy [21,65]. Regarding user experience, the participant responses were comparable to those reported in the previous research on similar concepts [23,31].

BCI was found to be more fatiguing according to the mVAS results. However, it is important to note that the evaluation runs for BCI were preceded by a calibration run, which could also induce fatigue. Participants indicated that the calibration procedure was the most cognitively demanding task of the study, which is in line with the fact that vigilance tasks with low information load are more fatiguing than higher-demand tasks [67]. Therefore, it is unclear whether the high fatigue levels were due to the BCI control system itself or the calibration process. Moreover, people often confuse mental fatigue with boredom and sleepiness, which are common responses to the repetitive nature of the calibration task [51].

The strengths of this study include its flexible procedure, which accommodates the iterative nature of software development and can be applied to any type of BCI control system. The comprehensive assessment of usability measures using realistic evaluation tasks ensures the relevance of the results for real-world applications. Furthermore, the control system’s portability is a notable strength, as all devices used are portable, making in-field evaluation studies trivial.

A notable weakness of the user study is the small sample size. Additionally, the limited number of sessions does not guarantee the participants’ optimal performance, and the effect of training was not directly assessed. Another limitation is that the study was not conducted with the target population of stroke patients. However, the current research suggests that BCI decoding models can be transferred from able-bodied individuals to stroke patients [68], and our previous research on the effects of neuroplasticity on BCI decoding performance in individuals with lower limb amputation [69] showed that there is no significant change in decoding performance between decoding models trained on data from able-bodied individuals and individuals with a lower limb amputation.

There are several enhancements available for the BCI control system. Increased efficiency without compromising effectiveness could be achieved through software optimizations aimed at improving the decision accuracy of the system. The potential optimizations include examining the chunk size when sampling EEG data and increasing the required number of predictions to make a decision. The former would enhance filtering by using a longer segment of EEG data, while the latter could prevent erroneous decisions caused by a few consecutive incorrect predictions. Studies investigating these specific aspects would, therefore, be worthwhile future work.

Better pre-processing beyond the currently limited filtering could improve decoding accuracy [70]. However, such modifications could lengthen decoding times, potentially reducing system efficiency. Using advanced decoding methods, such as deep learning, could also enhance decoding accuracy [13]. However, these methods often require large amounts of data and take longer to train before reaching optimal performance. Promising methods that improve decoding while addressing the need for more training data include transfer learning [71], continual learning [72], and novel data augmentation techniques such as diffusion models [73]. Benchmarks on the effect of including such methods are necessary to determine if it would be worth including these methods in a real-time EEG decoding pipeline.

Another potential improvement is to enhance the calibration and user training procedures through gamification. Observations during the user study suggested that users perform best when they intuitively think of the required movement as part of a familiar action, without over-concentrating on the movement itself. Previous research has shown that gamification boosts motivation and focus, which are essential for effective BCI use [66,74]. Making the calibration process more engaging could also reduce perceived fatigue and ensure that the data are collected at peak focus levels of the user. Therefore, training users through a game could help them familiarize themselves with MI and the control system in an immersive and motivating environment. Additionally, analyzing the recorded EEG data to identify markers of mental fatigue [75] could offer further insights into the extent to which the calibration procedure induces fatigue.

In the long term, one of our goals would be to integrate all hardware and software components of the BCI control system into a single, all-in-one device. This device could resemble an AR headset like the HoloLens 2, with built-in EEG sensors and potentially other useful sensors. Concepts already exist for such a device [76]. It would also need embedded computing hardware to ensure it is self-contained, comfortable, and privacy-guaranteed. Embedded EEG decoding is an active area of research [77,78].

To further validate the real-world usability of BCI control systems, the user study procedure could be expanded to include in-field studies where participants use the control system in their own homes. Such a study could investigate the long-term effects of the control system and periodically assess if usability evolves over time. This would also provide a suitable evaluation of proposed long-term machine learning methods such as continual learning.

## 6. Conclusions

Our findings indicate that the shared BCI control system is effective for task completion, demonstrating the feasibility of our shared control strategy in real-world settings. However, the current efficiency of BCI is inadequate for practical real-world applications, and the calibration process induces significant user fatigue.

While the system could be deployed in real-world conditions, our study shows that eye tracking offers superior usability for operating an assistive robotic arm. Currently, BCI is practical only for niche applications that require the user to maintain their gaze on a fixed point, such as in assembly tasks. Further advancements are needed to justify the increased hardware and software complexity.

We see substantial potential for improving the system’s reliability and efficiency through advanced AI methods like deep learning, which can enhance decoding accuracy and reduce user training time. We are confident that this would result in our approach surpassing the current state of the art in terms of usability. Additionally, gamifying the calibration process could reduce fatigue and enhance user experience. With improvements in decoding robustness and strategies to boost focus and mitigate calibration-induced fatigue, BCI could become a practical choice for real-world applications.

## Figures and Tables

**Table 1:** Mean and standard deviation of UEQ scores for BCI and Eye tracking control types with *p*-values for statistical tests.

Question	BCI	Eye Tracking	*p*-Value
annoying—enjoyable	5.00(±1.78)	6.08(±0.86)	0.052
not understandable—understandable	4.92(±1.75)	6.38(±0.77)	**0.019**
creative—dull	2.31(±1.25)	2.54(±1.66)	0.570
easy to learn—difficult to learn	4.00(±1.96)	1.85(±1.57)	0.008
valuable—inferior	2.85(±1.28)	1.77(±0.73)	0.005
boring—exciting	4.54(±1.66)	4.85(±1.52)	0.677
not interesting—interesting	5.85(±1.28)	6.31(±0.75)	0.139
unpredictable—predictable	4.00(±1.68)	5.38(±1.94)	0.050
fast—slow	3.69(±1.55)	2.85(±1.77)	0.027
inventive—conventional	2.08(±1.12)	2.00(±0.91)	1.000
obstructive—supportive	4.23(±2.01)	5.62(±0.96)	0.015
good—bad	2.92(±1.75)	1.69(±0.75)	0.014
complicated—simple	4.15(±1.77)	5.69(±1.65)	0.048
unlikable—pleasing	5.38(±1.04)	5.46(±0.97)	0.837
usual—leading edge	4.69(±1.84)	5.00(±1.73)	0.751
unpleasant—pleasant	4.31(±1.60)	5.23(±1.24)	0.165
secure—not secure	3.54(±1.66)	2.69(±1.44)	0.020
motivating—demotivating	3.62(±1.76)	2.46(±1.13)	0.050
meets expectations—does not meet expectations	3.62(±2.02)	2.00(±1.35)	0.060
inefficient—efficient	4.31(±1.60)	5.77(±1.17)	0.014
clear—confusing	3.15(±1.68)	2.00(±1.15)	0.046
impractical—practical	4.77(±1.48)	5.54(±1.13)	0.165
organized—cluttered	2.38(±1.26)	2.23(±1.01)	0.549
attractive—unattractive	2.77(±1.09)	2.46(±1.45)	0.596
friendly—unfriendly	2.85(±1.34)	2.15(±1.07)	0.069
conservative—innovative	6.00(±0.82)	5.77(±0.93)	0.337

## Data Availability

The data presented in this study are available on request from the corresponding author due to the highly personal nature of the data.

## Appendix A. User Experience Interview

**Did you feel like you were able to successfully complete the tasks with the BCI control system?**
**Did you feel like you were in control of the robotic arm?**
**Do you think that BCI provides an added value to the control system, assuming that you are not able to move or talk?**
**Which control system variant did you prefer? (Only when eye tracking and BCI were compared)**
**Do you think a different approach would work better? If yes, what do you suggest?**
**Do you have any suggestions for improving the current control system?**
